# Association between eating alone and depressive symptom in elders: a cross-sectional study

**DOI:** 10.1186/s12877-016-0197-2

**Published:** 2016-01-16

**Authors:** Xinyi Wang, Wei Shen, Chunmei Wang, Xiaoyi Zhang, Yuanyuan Xiao, Fan He, Yujia Zhai, Fudong Li, Xiaopeng Shang, Junfen Lin

**Affiliations:** Zhejiang Provincial Centre for Disease Control and Prevention, 3399 Binsheng Road, Binjiang District, Hangzhou, Zhejiang Province China; Tongxiang Centre for Disease Control and Prevention, 64 East Maodun Road, Wutong Subdistrict, Tongxiang, Zhejiang Province China; School of Public Health, Kunming Medical University, 1168 Yuhua Road, Chenggong District, Kunming, Yunnan China

**Keywords:** Eating alone, Depressive symptom, Elder, Living alone

## Abstract

**Background:**

Depression is one of the main health concerns in elders which could lead to many negative outcomes. Eating alone is an emerging issue in elders in China and would become more serious along with the fast course of industrialization and urbanization, as well as population aging. However, their relationship was seldom researched.

**Methods:**

Using a two-stage cluster sampling strategy, an investigation was conducted in 2014 among 9,361 permanent residents aged 60 years and above in Zhejiang Province, China. Totally, 7,968 cognitively normal elders were included in our analysis. Multilevel logistic regression was used to explore the association between eating arrangement (number of companions in mealtime) and depressive symptom as well as the association between eating alone and depressive symptom in relation to living alone.

**Results:**

In our sample, 17.1 % of the elders ate alone and 8.7 % had depressive symptom. We observed a distinct inverse association between eating arrangement (number of companions in mealtime) and geriatric depressive symptom (female: OR = 1.12, P = 0.027; aged 60–74 years: OR = 1.16, P = 0.002), after adjustment for demography, socioeconomic status, social relation, health behaviors, underlying conditions and living alone. We then introduced a combined variable of eating alone and living alone and examined its relationship with depressive symptom. We found that compared with elders who ate and lived with others, those who ate alone but lived with others had a significant increased odds of depressive symptom (female: OR = 1.62, P = 0.027; aged 60–74 years: OR = 1.59, P = 0.025).

**Conclusions:**

Our results suggested that a lack of companionship in mealtime might be independently associated with geriatric depressive symptom for females and those aged 60–74 years. What’s more, eating alone might be a stronger factor associated with depressive symptom than living alone. We recommend interventions to be performed in encouraging elders especially females and those aged 60–74 years to eat with others. Longitudinal studies in different populations which focus on this topic are required to better understand this issue.

## Background

One of the main health problems in the elderly population is depression, of which the rate lied between 4.6 and 9.3 % in terms of major depression, and varied from 4.5 to 47.5 % when measured by depressive symptom [1–4]. Depression could lead to many negative outcomes such as disability, ischemic heart disease and even death [5, 6], greatly affecting life quality of elders and increasing health care cost as well [7].

Several risk factors were found to be related with depression, such as female gender, old age, low-level education, living alone, loneliness and poor social support [1, 3, 4, 8–11]. However, association on eating arrangement and depression has so far attracted little attention.

Dining is a very important occasion in daily social life in China and many other countries. People who eat meals with others are expected to be benefited from social support in shared mealtime while those who eat alone fail to have this advantage. Eating alone was found to be related with calorie intake, food diversity and overweight [12, 13]. What’s more, a Japanese study found that in elders in Tosa town, those who ate alone had statistically higher scores of depression than those who ate with others, based on the 15-item Geriatric Depression Scale. Further multivariate analysis for this data revealed that eating alone was statistically associated with depression [14]. Another recently published Japanese study also showed that eating alone was a strong factor associated with depressive symptom in elders. Among elders aged 65-74 years and elders aged above 74 years in Kashiwa city, odds ratios of depressive symptom for those who lived with others yet ate alone was 5.02 and 2.41, respectively, compared to their counterparts who lived and ate with others [15]. However, both of the studies were conducted in a confined region in Japan. The evidence on association between eating alone and depression/depressive symptom is very limited in other ethnic or geographical populations.

Like many countries in the rest of the world, China is facing an emerging concern that a large group of elders are now eating alone. What is more, this population would probably further expand in the future due to population aging as well as industrialization and urbanization. Under this circumstance, a better understanding on the relationship between eating alone and geriatric depressive symptom is warranted. Using data from a large-scale investigation in elders in Zhejiang Province, we aimed to explore this association here in the study. Besides, we combined the information of eating alone and living alone, to further discuss the association between eating alone and depressive symptom in relation to living alone.

## Methods

### Data source

Data were collected in 2014 in six randomly selected counties/districts among a total of 90 in Zhejiang Province, an economically developed province in eastern China. For five counties/districts, one town/subdistrict was further randomly selected in each of them. For one county, which is located in a mountainous area, two towns were randomly selected instead, due to its sparse population. All permanent residents aged 60 years old and above in these selected town/subdistrict were then invited to participate in the investigation. The investigation was approved by the Ethics Committee of Zhejiang Provincial Centre for Disease Control and Prevention. A written consent was acquired from each of the participant. For those who were illiterate, the content was read by the interviewer and signed with the participant’s finger print.

Participants were interviewed face to face based on a self-designed questionnaire by an experienced and previously trained public health practitioner or nurse in a local centre for disease control and prevention or health service centre. Their information of demography, socioeconomic status, social relation, health conditions and behaviors, activity of daily living, cognitive function, living and eating arrangement, food consumption, reproductive history and depressive symptoms was collected. Data were entered by the interviewers and checked by staff at Zhejiang Provincial Centre for Disease Control and Prevention. Missing data and logical errors were fed back to the initial interviewer who would try to reinvestigate the participants to complete the dataset. Totally, 9,361 elders aged 60 years and above responded to the investigation, with a response rate of 76.0 %.

For the 9,361 elders participating in the investigation, those whose information pertaining to any of the following situations were excluded in our analysis: (1) absence of answer in the question of number of persons eating meals together, (2) absence of answers in three or more items in the questions related to depressive symptoms and (3) presence of cognitive impairment. Cognitive function was examined by the mini-mental state examination (MMSE), with a greater score indicating a better cognitive function. Those who were (1) illiterate and scored less than 18, (2) educated in primary school level and scored less than 21 or (3) educated in middle school level or higher and scored less than 25, were considered as cognitive impairment [16].

Finally, 1,393 out of 9,361 participants were excluded, among whom 33 reported no answer to questions of depression or eating arrangement, and 1,360 were considered as cognitive impairment.

### Variables of interest

Depressive symptoms were examined by the Patient Health Questionnaire-9 (PHQ-9). Participants were asked by the question of how often in the past two weeks were they troubled by the nine depressive symptoms described in the PHQ-9. Each question had a four-category answer (PHQ score 0: never, PHQ score 1: less than a week, PHQ score 2: more than a week, PHQ score 3: nearly every day in the two weeks). Levels of depressive symptom severity were defined as follows: total PHQ score 0 to 4 equals to none depressive symptoms and total PHQ score 5 to 27 equals to presence of depressive symptoms [17, 18].

Eating arrangement (number of companions in mealtime) was measured by the question of how many people were there in your house having meals with you in the last year. Answers were categorized into five groups which were (1) four persons and more, (2) three persons, (3) two persons, (4) one person and (5) eating alone, respectively.

### Covariates

Demographic factors (e.g. age, gender and ethnicity) [8, 19–21], socioeconomic status (e.g. education, employment status and economic level) [2, 8, 19, 22–26], social relation (e.g. marriage and number of children) [27–30], health behaviours (e.g. physical activity, drinking and smoking,) [28, 31, 32], medical conditions [19, 20, 23, 25, 33], living alone [9, 23, 34] and limitation in activity of living [35] were found to be related to depressive symptom/depression in previous literatures. Therefore, we introduced relevant data in our analysis and treated the following variables as covariates, which were gender, age, education, employment status, perception of economic status, marriage, having a child, smoking, drinking, physical activity, underlying conditions, limitation in activity of daily living and living alone. Detailed definition of perception of economic status, underlying conditions, limitation in activity of daily living and living alone were described as follows.

Perception of economic status was asked in the question of in which level you thought your income was in your residential area. Answers were categorized into three categories which were (1) poor, (2) median and (3) rich. Presence of underlying conditions was identified by participants’ self-report of any of the following diagnoses which were (1) hypertension, (2) diabetes, (3) coronary heart diseases, (4) respiratory diseases and (5) tumors/cancers. Limitation in activity of daily living was determined by the Elderly Activities of Daily Living Scale (EADL) in the Chinese National Standard of Basic Public Health Service [36]. Five activities were included in the EADL, which were eating, bathing, dressing, toilet hygiene and functional mobility. Participants who gave answers of self-dependent to all the above activities were considered as having no limitation in activity of daily living, otherwise were regarded as having limitation in it. Living alone was asked by the question of how many people were there living together with you. Answers were categorized into two groups which were (1) living alone and (2) not alone, respectively.

### Data analysis

Variables relevant to demography, socioeconomic status, social relation, health behaviours, medical conditions, living arrangement and activity of daily living were extracted from our dataset and each of them was examined on its association with eating arrangement and with depressive symptom as well. Chi-square tests or Chi-square tests for trend, as appropriate, were used for this univariate analysis. Those variables showing statistical associations with both eating arrangement and depressive symptom, and were not in the pathway between them were regarded as covariates for this association. The covariates were further categorized into six groups which were (1) gender and age, (2) education, employment status and perception of economic status, (3) marriage and having a child, (4) smoking, drinking and physical activity, (5) underlying conditions and (6) living alone.

Taking county/district difference of depressive symptom into account, multilevel logistic regression with random intercept was applied to explore the association between eating arrangement and depressive symptom, adjusted for the six groups of covariates step by step.

To understand the association between eating alone and depressive symptom in relation to living alone, a four-category variable combining information of eating alone and living alone was further introduced by using multilevel logistic analysis. Limitation in activity of daily living was additionally included as a covariate in this model as it met the covariate selection criteria mentioned above.

Based on the previous report of gender and age variance in depression/depressive symptom [1, 19], all models were stratified by gender and age (aged 60–74 years and aged 75 years and above).

Data were analyzed using STATA SE 12. All the statistical tests we reported were two-sided, and *P* < 0.05 was considered as statistically significant.

## Results

Details of study participants’ information were shown in Table 1. The median age of the participants included in the analysis was 67 years old. The gender ratio (male: female) was 1.02. The prevalence of depressive symptom was 8.7 %. The percentage of participants who ate alone was 17.1 % and that of participants who lived alone was 14.5 %. A trend was observed in age group associated with depressive symptom (*P* < 0.001). Another trend was seen in eating arrangement associated with depressive symptom (*P* < 0.001). All factors excepting for ethnicity and limitation in activity of daily living were shown to be statistically associated with both depressive symptom and eating arrangement based on Chi square tests (*P* < 0.05).

**Table 1:** Characteristics of study participants

Characteristics		N	%	Depressive symptom (%)	P^*^
Gender	Male	4015	50.4	6.7	<0.001
	Female	3953	49.6	10.8	
Age	60−	2927	36.7	7.8	<0.001
	65−	2067	25.9	7.6	
	70−	1280	16.1	9.5	
	75−	947	11.9	11.2	
	80+	747	9.4	11.0	
Ethnicity	Han ethnicity	7716	96.9	8.7	0.711
	Minorities	245	3.1	9.4	
Education	Illiteracy	3819	48.0	9.7	0.017
	Primary school	3539	44.4	7.9	
	Middle school or Higher	605	7.6	7.4	
Perception of	Rich	816	10.3	2.8	<0.001
economic status	Moderate	6274	78.8	7.9	
	Poor	869	10.9	20.3	
Employment status	Working	3199	40.3	7.4	<0.001
	Retired	4278	53.8	9.9	
	Never work	469	5.9	6.8	
Marriage	Single	118	1.5	13.6	<0.001
	Married	6167	78.1	7.9	
	Widowed/divorced	1610	20.4	11.4	
Having a child	Yes	7787	97.8	8.6	0.010
	No	177	2.2	14.1	
Smoking	Currently yes	1814	22.8	6.1	<0.001
	Quitted	800	10.0	9.1	
	Never	5353	67.2	9.6	
Drinking	Currently yes	2135	26.8	5.9	<0.001
	Quitted	703	8.8	11.4	
	Never	5129	64.4	9.6	
Physical activity	Yes	1553	19.5	12.4	<0.001
	No	6409	80.5	7.9	
Underlying conditions	Yes	4045	50.8	10.2	<0.001
	No	3923	49.2	7.2	
Limitation in activity	Yes	203	2.6	29.1	<0.001
of daily living	No	7731	97.4	8.2	
Eating arrangement	With 4 persons or more	1382	17.3	4.5	<0.001
	With 3 persons	748	9.4	7.8	
	With 2 persons	758	9.5	6.7	
	With 1 person	3718	46.7	9.4	
	Eating alone	1362	17.1	12.9	
Living alone	Living alone	1152	14.5	12.2	<0.001
	Not alone	6807	85.5	8.1	

^*^Chi-square tests for trend were used to examine the association of depressive symptom with age and eating arrangement, respectively. Chi-square tests were used to examine the association between depressive symptom and each of the rest variables

Gender-specific multilevel logistic regression models of association between eating arrangement and depressive symptom were shown in Table 2. In model A where only age was controlled, a significant association was seen for both genders (male: OR = 1.19, *P* = 0.005; female: OR = 1.13. *P* = 0.010), indicating that decreased number of companions in mealtime were associated with an increased odds of depressive symptom. When additionally adjusted for socioeconomic status (education, employment status and perception of economic status) in model B, OR decreased slightly for both genders (male: OR = 1.15, *P* = 0.019; female: OR = 1.10. *P* = 0.032), showing a weaker association. Further adjustment for marriage and having a child in model C seldom changed the association (male: OR = 1.15, *P* = 0.030; female: OR = 1.11. *P* = 0.035). Similarly, no obvious change was observed for both genders when additionally adjusted for health behaviors (smoking, drinking and physical activity) in model D and underlying conditions in model E, respectively. Finally in model F when additionally adjusted for living alone which was thought to be a strong confounder, the association for female remained significant (OR = 1.12, *P* = 0.027) while it failed to reach the statistical significance for male (OR = 1.14, *P* = 0.052).

**Table 2:** Multilevel logistic regression models of association between eating arrangement and depressive symptom by gender

Model^a^	Male				Female			
	OR	CI	P^h^	P^i^	OR	CI	P^h^	P^i^
Model A^b^	1.19	(1.05,1.33)	0.005	<0.0001	1.13	(1.03,1.23)	0.010	<0.0001
Model B^c^	1.15	(1.02,1.30)	0.019	<0.0001	1.10	(1.01,1.21)	0.032	<0.0001
Model C^d^	1.15	(1.01,1.30)	0.030	<0.0001	1.11	(1.01,1.21)	0.035	<0.0001
Model D^e^	1.14	(1.01,1.29)	0.035	<0.0001	1.11	(1.01,1.22)	0.031	<0.0001
Model E^f^	1.14	(1.01,1.29)	0.036	<0.0001	1.11	(1.01,1.22)	0.030	<0.0001
Model F^g^	1.14	(1.00,1.29)	0.052	<0.0001	1.12	(1.01,1.24)	0.027	<0.0001

^a^For all the multilevel logistic regression models in this table, the variable of county/district was treated as the group variable in multilevel logistic regression

^b^Model A was the basic model and only adjusted for age

^c^Model B was additionally adjusted for education, employment status and perception of economic status

^d^Model C was additionally adjusted for marriage and having a child

^e^Model D was additionally adjusted for smoking, drinking and physical activity

^f^Model E was additionally adjusted for underlying conditions

^g^Model F was finally adjusted for living alone

^h^The P value was obtained by the multilevel logistic regression for the association between eating arrangement and depressive symptom

^i^The P value was obtained by the likelihood ratio test comparing multilevel logistic regression model with logistic regression model for the association between eating arrangement and depressive symptom

Age-specific multilevel logistic regression models of association between eating arrangement and depressive symptom were shown in Table 3. For elders aged 60–74 years, model A (adjusted for gender only) showed a distinct inverse association between eating arrangement and depressive symptom (OR = 1.17, *P* < 0.001). When additionally adjusted for socioeconomic status in model B, OR decreased to 1.14 (*P* = 0.001). In model C, model D, model E and model F, where social relation, health behaviours, underlying conditions and living alone were introduced successively, the association did not change obviously and still remained significant (for model F: OR = 1.16, *P* = 0.002). However, for those aged 75 years and above, all the adjusted models failed to reach the statistical significance (*P* > 0.05).

**Table 3:** Multilevel logistic regression models of association between eating arrangement and depressive symptom by age

Model^a^	Aged 60–74				Aged 75+			
	OR	CI	P^h^	P^i^	OR	CI	P^h^	P^i^
Model A^b^	1.17	(1.08,1.27)	<0.001	<0.0001	1.10	(0.95,1.27)	0.205	<0.0001
Model B^c^	1.14	(1.05,1.24)	0.001	<0.0001	1.06	(0.91,1.22)	0.467	<0.0001
Model C^d^	1.15	(1.06,1.26)	0.001	<0.0001	1.03	(0.89,1.19)	0.714	<0.0001
Model D^e^	1.15	(1.05,1.25)	0.002	<0.0001	1.04	(0.89,1.20)	0.647	<0.0001
Model E^f^	1.15	(1.05,1.25)	0.002	<0.0001	1.04	(0.89,1.20)	0.624	<0.0001
Model F^g^	1.16	(1.06,1.26)	0.002	<0.0001	1.01	(0.86,1.18)	0.939	<0.0001

^a^For all the multilevel logistic regression models in this table, the variable of county/district was treated as the group variable in multilevel logistic regression

^b^Model A was the basic model and only adjusted for gender

^c^Model B was additionally adjusted for education, employment status and perception of economic status

^d^Model C was additionally adjusted for marriage and having a child

^e^Model D was additionally adjusted for smoking, drinking and physical activity

^f^Model E was additionally adjusted for underlying conditions

^g^Model F was finally adjusted for living alone

^h^The P value was obtained by the multilevel logistic regression for the association between eating arrangement and depressive symptom

^i^The P value was obtained by the likelihood ratio test comparing multilevel logistic regression model with logistic regression model for the association between eating arrangement and depressive symptom

Gender- and age-specific information of elders eating alone and living alone were shown in Table 4. Tested by the univariate analysis, a prominent association was observed between eating alone and depressive symptom in relation to living alone, for both genders and age groups (*P* < 0.05).

**Table 4:** Gender- and age-specific univariate association between eating alone and depressive symptom in relation to living alone

Characteristic		N	%^a^	%^b^	Depressive symptom (%)	P^c^
Male	Both not alone^d^	3351	83.52		6.03	<0.001
	Eating alone only^e^	205	5.11	5.76	10.24	
	Living alone only^f^	89	2.22		3.37	
	Both alone^g^	367	9.15		11.17	
Female	Both not alone	3041	77.05		9.70	<0.001
	Eating alone only	210	5.32	6.46	16.67	
	Living alone only	117	2.96		16.24	
	Both alone	579	14.67		13.47	
Aged 60–74	Both not alone	5303	84.60		7.49	<0.001
	Eating alone only	280	4.47	5.02	13.21	
	Living alone only	153	2.43		9.87	
	Both alone	533	8.50		10.88	
Aged 75+	Both not alone	1089	64.40		9.18	0.011
	Eating alone only	135	7.98	11.03	14.07	
	Living alone only	54	3.19		12.96	
	Both alone	413	24.42		14.77	

^a^Percentage of participants in the entire sample

^b^Percentage of participants in those who lived with others

^c^The P value was obtained by chi square test

^d^Both not alone: eating and living with others

^e^Eating alone only: eating alone but living with others

^f^Living alone only: living alone but eating with others

^g^Both alone: eating alone and living alone

**Fig. 1:**
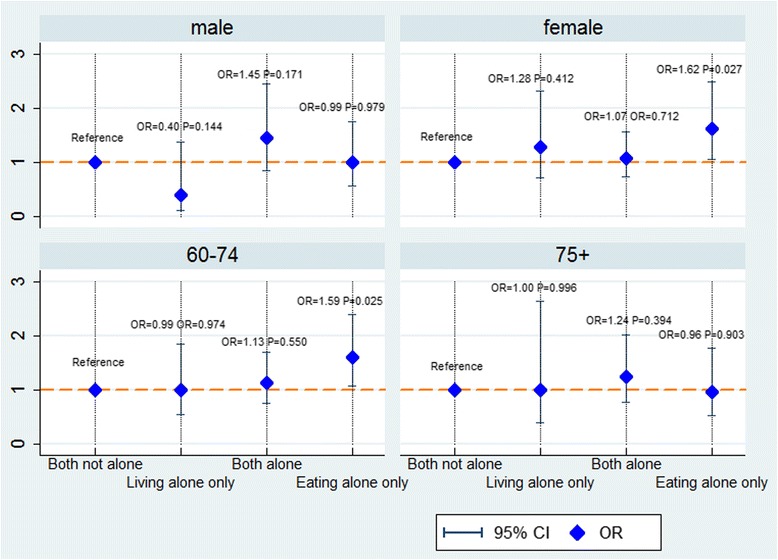
Gender- and age-specific multilevel logistic regression of association between eating alone and depressive symptom in relation to living alone. Gender- and age-specific multilevel logistic regression model was used to analyse the association between eating alone and depressive symptom in relation to living alone, adjusted for age, gender, education, employment status, perception of economic status, marriage, having a child, smoking, drinking, physical activity, underlying conditions and limitation in activity of daily living. The variable of county/district was treated as the group variable in the multilevel logistic regression model. For each of the four associations displayed in the figure, multilevel logistic regression model was shown to be an improved one compared with logistic regression model, examined by the likelihood ratio test (*P* < 0.001). Both not alone: eating and living with others. Living alone only: living alone but eating with others. Both alone: eating and living alone. Eating alone only: eating alone but living with others

Gender- and age-specific multilevel logistic regression models for the association between eating alone and depressive symptom in relation to living alone were presented in Fig. 1. For elderly females, those who ate alone but lived with others had the highest odds of depressive symptom (OR = 1.62, *P* = 0.027), compared to those who ate and lived with others (OR = 1.00, reference), those who lived alone but ate with others (OR = 1.28, *P* = 0.412) and those who ate and lived alone (OR = 1.07, *P* = 0.712). Similar pattern was seen for elders aged 60–74 years. Those who ate alone but lived with others had the highest odds of depressive symptom (OR = 1.59, *P* = 0.025), compared to the other three groups of their counterparts. For both elderly males and those aged 75 years and above, no statistical significant result was observed for the association between eating alone and depressive symptom in relation to living alone (*P* > 0.05).

For each association displayed in Table 2, Table 3 and Fig. 1, the multilevel logistic regression model with random intercept was shown to be an improved one compared with the logistic regression model, tested by likelihood ratio test (*P* < 0.001).

## Discussion

The prevalence of depressive symptom among cognitively normal elders in this cross- sectional study was 8.7 %. This was much lower than those reported in most of the previous studies, which were around 20 % and even higher [19, 37, 38]. The gaps could be largely attributed to the exclusion of elders with cognitive impairment in our analysis, measures of depressive symptoms and differences in population and socio-culture contexts as well. There were 17.1 % elders eating daily meals alone in our sample, higher than the number of living alone (14.5 %). This result accords with our expectation that a non-ignorable group of elders in Zhejiang Province is suffering from eating daily meals alone.

We found a distinct inverse association between eating arrangement (number of companions in mealtime) and depressive symptom, after adjustment for factors like demography, socioeconomic status, social relation, health behaviors, underlying conditions and living arrangement. Similar evidence was found in a Japanese study conducted among 856 elderly people, which revealed that depression was statistically associated with eating alone after adjustment for age, gender, BMI and food diversity (OR = 1.42, 95 % CI:1.00–2.11, *P* = 0.043) [14]. Other evidence on this topic is yet very rare. Dining is a very important occasion of daily social life in Asia and many other countries, where family members and close ones are supposed to gather together sharing food and emotion. In Chinese tradition, eating with several people (family members, friends and so on) at home is always considered to be “Re Nao”, which means bustling, lively and harmonious. People who eat alone are supposed to get less social support and interaction than those who eat with others. Previous studies in both western and eastern countries have found that loneliness and lack of social support were associated with depression, with feeling of loneliness or lack of social support increasing the likelihood of depression or depressive symptom [3, 10, 11, 39–41]. Researcher also found that daily hassles were stronger predictors of psychological symptoms than major life events [42]. Therefore, the association between eating arrangement and depressive symptom we found here could be to some extent attributed to the accumulated negative impact of lonely feeling and social isolation in daily mealtime.

Dislike living arrangement, eating arrangement is not confined by the housing condition: people who live together might choose to eat separately because of different daily schedules or conflicts within the family, whereas people who live separately might eat together because of their willingness to gather and communicate. In our sample, 5.2 % participants ate alone but lived with others. Among those who lived with others, 6.1 % ate alone. Currently, only very few studies looked into the association between eating alone and depressive symptom in relation to living alone. A recent paper displayed an intriguing result that among a sample of 1,856 elders aged 65–94 years in Kashiwa city in Japan, elders who ate alone but lived with others were at the highest risk of exhibiting depressive symptom (64-74: OR = 5.02; 75+: OR = 2.41), when compared with their counterparts who ate and lived with others [15]. We observed a similar result here in this study: compared with those who ate and lived with others, those who lived alone but ate with others and those who ate and lived alone, elders who ate alone but lived with others had the highest odds of depressive symptom. Speculating from this, eating alone might be a stronger factor associated with depressive symptom than living alone. Meanwhile, it was an interesting result that elders who ate alone but lived with others had an even higher odds of depressive symptom than those who ate and lived alone. Explanation to this phenomenon is difficult to make due to the absence of detailed information on eating and living arrangement. One plausible reason we try to surmise is that elders who live alone might get used to eating alone, but elders who live with others might expect to eat with them as well. Therefore, when they fail to do so in reality, they gain more disappointment and loneliness, which might deteriorate mental health. What’s more, combative relationship and housing constraint might be the main reason for elders living with others but eating alone. The negative effect of conflict within family might neutralize or even outweigh the benefit of coresidence, and thus might result in a higher odds of depressive symptom than those who ate and lived alone.

Our stratified analysis showed that both the association between eating arrangement and depressive symptom and the one between eating alone and depressive symptom in relation to living alone remained significant in elderly females and elders aged 60-74 years. In the present sample, prevalence of depressive symptom was higher in females than in males, and increased with age, which was coincident with many of the previous studies documenting gaps of depression in gender and age [1, 19]. Compared to men, women are at greater mortality risk when they are in poor social network, which indicates that women might be more dependent on companionship to gain health [43]. Hence we could speculate that a lack of companionship during eating daily meals would affect women’s mental health more greatly than men, which was seen in our analysis.

As for age, an interesting result was seen in our analysis that the association exits in elders aged younger than 75 years rather than those older than 75 years. Some literatures have stated that depressive symptoms might be experienced differently between younger and older adults. It was characterized more of psychological conditions for the younger adults while for the older ones somatic conditions were more prevalent, such as memory complaints and poor sleep quality [1, 44, 45]. Therefore, psychological support such as companionship during eating daily meals might logically help relieve depression in younger elders but not in older ones, as the older ones suffered mainly from somatic symptoms associated with depression. Meanwhile, it could be expected that older elders might have a better adaptation to the situation of being alone due to its longer experience of aging process. They are probably in the condition of being alone longer than the younger ones, and had more time of identity balance which could play a great role in helping them gradually get used to the social isolation. In other words, older elders are not as much sensitive to social support as the younger ones. A similar standpoint was described in a review of depression in elders which pointed that social risk such as poor social support were not as prominent in older adults as in younger ones [22]. Thus the association between depressive symptom and social support like mealtime companionship might be only seen in the younger elders. However, understanding of the result we found here was limited since only a few literatures were retrieved digging deep the age difference in depression. Hence other explanations could not be ruled out. Further in-depth research on the age difference in depressive symptom and its association with eating arrangement is needed.

This data analysis was based on a well-designed study with a large sample size, which brought stability and reliability to results. However, we still have several limitations. First, data were from a cross-sectional survey, thus no causal effect conclusion can be made. However, we measured depressive symptom in the past two weeks while measured eating arrangement in the past year, therefore the temporal relation of the association is more likely to be from eating arrangement to depressive symptom than the opposite way. Second, as a consequence of a retrospective study, recall bias is difficult to avoid. However, participants with cognitive impairment were excluded in our analysis, which at least prevented serious recall bias. Third, our data were drawn form a study of health aging instead of a panel research of eating arrangement and depression. Therefore details of eating arrangement such as who are the companions and information of other potential confounder such as social support are not available. Relevant questions have been added in the questionnaires in 2015, which is expected to explore the association at a much deeper level.

## Conclusions

This paper displayed one of the first attempts to explore the gender- and age-specific association between eating alone and depressive symptom in elders as well as its relation to living alone. Our results suggested that a lack of companionship in mealtime might be independently associated with geriatric depressive symptom for females and those aged 60-74 years. What’s more, eating alone might be a stronger factor associated with depressive symptom than living alone. Dislike suggestion of living with others, eating with others is a more feasible intervention that could be introduced in public health action. We hereby suggest that interventions should be performed to encourage elders especially female and those aged 60-74 years to eat with others such as family members, friends and neighbours. Longitudinal studies in different populations which focus on this topic are required to better understand this issue.

## References

[1] Luppa M, Sikorski C, Luck T, Ehreke L, Konnopka A, Wiese B (2012). Age- and gender-specific prevalence of depression in latest-life--systematic review and meta-analysis. J Affect Disord.

[2] Assil SM, Zeidan ZA (2013). Prevalence of depression and associated factors among elderly sudanese: a household survey in Khartoum State. East Mediterr Health J.

[3] Ng CW, Tan WS, Gunapal PP, Wong LY, Heng BH (2014). Association of Socioeconomic Status (SES) and Social Support with Depressive Symptoms among the Elderly in Singapore. Ann Acad Med Singap.

[4] Fukunaga R, Abe Y, Nakagawa Y, Koyama A, Fujise N, Ikeda M (2012). Living alone is associated with depression among the elderly in a rural community in Japan. Psychogeriatrics.

[5] Charlson FJ, Moran AE, Freedman G, Norman RE, Stapelberg NJ, Baxter AJ (2013). The contribution of major depression to the global burden of ischemic heart disease: a comparative risk assessment. BMC Medicine..

[6] Blazer DG (2003). Depression in late life: review and commentary. J Gerontol Ser A Biol Med Sci.

[7] Luppa M, Heinrich S, Angermeyer MC, Konig HH, Riedel-Heller SG (2007). Cost-of-illness studies of depression: a systematic review. J Affect Disord.

[8] Sengupta P, Benjamin AI (2015). Prevalence of depression and associated risk factors among the elderly in urban and rural field practice areas of a tertiary care institution in Ludhiana. Indian J Public Health.

[9] Chan A, Malhotra C, Malhotra R, Ostbye T (2011). Living arrangements, social networks and depressive symptoms among older men and women in Singapore. Int J Geriatr Psychiatry.

[10] Aylaz R, Akturk U, Erci B, Ozturk H, Aslan H (2012). Relationship between depression and loneliness in elderly and examination of influential factors. Arch Gerontol Geriatr.

[11] Chao SF (2011). Assessing social support and depressive symptoms in older Chinese adults: a longitudinal perspective. Aging Mental Health.

[12] Locher JL, Robinson CO, Roth DL, Ritchie CS, Burgio KL (2005). The effect of the presence of others on caloric intake in homebound older adults. J Gerontol Ser A Biol Med Sci.

[13] Hammons AJ, Fiese BH (2011). Is frequency of shared family meals related to the nutritional health of children and adolescents?. Pediatrics.

[14] Kimura Y, Wada T, Okumiya K, Ishimoto Y, Fukutomi E, Kasahara Y (2012). Eating alone among community-dwelling Japanese elderly: association with depression and food diversity. J Nutr Health Aging.

[15] Kuroda A, Tanaka T, Hirano H, Ohara Y, Kikutani T, Furuya H (2015). Eating Alone as Social Disengagement is Strongly Associated With Depressive Symptoms in Japanese Community-Dwelling Older Adults. J Am Med Dir Assoc..

[16] Zeng G (1995). Aging of brain and Alzheimer disease (in Chinese).

[17] Association AP: Severity Measure for Depression—Adult (Patient Health Questionnaire [PHQ-9]). In: American Psychiatric Association.

[18] Spitzer RL, Kroenke K, Williams JB (1999). Validation and utility of a self-report version of PRIME-MD: the PHQ primary care study. Primary Care Evaluation of Mental Disorders. Patient Health Questionnaire. JAMA.

[19] Glaesmer H, Riedel-Heller S, Braehler E, Spangenberg L, Luppa M (2011). Age- and gender-specific prevalence and risk factors for depressive symptoms in the elderly: a population-based study. Int Psychogeriatr.

[20] Djernes JK (2006). Prevalence and predictors of depression in populations of elderly: a review. Acta Psychiatr Scand.

[21] Margaretten M, Barton J, Julian L, Katz P, Trupin L, Tonner C (2011). Socioeconomic determinants of disability and depression in patients with rheumatoid arthritis. Arthritis Care Res.

[22] Fiske A, Wetherell JL, Gatz M (2009). Depression in older adults. Annu Rev Clin Psychol.

[23] Lai DW (2004). Depression among elderly Chinese-Canadian immigrants from Mainland China. Chin Med J.

[24] Imai H, Chen WL, Fukutomi E, Okumiya K, Wada T, Sakamoto R (2015). Depression and subjective economy among elderly people in Asian communities: Japan, Taiwan, and Korea. Arch Gerontol Geriatr.

[25] Hovaldt HB, Andersen I, Sandager M, Sperling C, Frederiksen K, Bidstrup PE (2015). The role of social position in anxiety and depressive symptoms among Danish cancer survivors. Acta Oncol.

[26] Wang L, Song R, Chen Z, Wang J, Ling F (2015). Prevalence of depressive symptoms and factors associated with it in type 2 diabetic patients: a cross-sectional study in China. BMC Public Health.

[27] Papadopoulos FC, Petridou E, Argyropoulou S, Kontaxakis V, Dessypris N, Anastasiou A (2005). Prevalence and correlates of depression in late life: a population based study from a rural Greek town. Int J Geriatr Psychiatr.

[28] Sapranaviciute-Zabazlajeva L, Reklaitiene R, Tamosiunas A, Baceviciene M, Virviciute D, Peasey A (2014). Correlates of depressive symptoms in urban middle-aged and elderly Lithuanians. Soc Psychiatry Psychiatr Epidemiol.

[29] Ambo H, Meguro K, Ishizaki J, Shimada M, Yamaguchi S, Sekita Y (2001). Depressive symptoms and associated factors in a cognitively normal elderly population: the Tajiri Project. Int J Geriatr Psychiatry.

[30] Al-Salmani A, Juma T, Al-Noobi A, Al-Farsi Y, Jaafar N, Al-Mamari K (2015). Characterization of depression among patients at urban primary healthcare centers in Oman. Int J Psychiatry Med.

[31] Kim SA, Kim E, Morris RG, Park WS (2015). Exploring the non-linear relationship between alcohol consumption and depression in an elderly population in Gangneung: the Gangneung Health Study. Yonsei Med J.

[32] Davis TM, Hunt K, Bruce DG, Starkstein S, Skinner T, McAullay D (2015). Prevalence of depression and its associations with cardio-metabolic control in Aboriginal and Anglo-Celt patients with type 2 diabetes: the Fremantle Diabetes Study Phase II. Diabetes Res Clin Pract.

[33] Kader Maideen SF, Sidik SM, Rampal L, Mukhtar F (2014). Prevalence, associated factors and predictors of depression among adults in the community of Selangor, Malaysia. PloS ONE.

[34] Oh DH, Park JH, Lee HY, Kim SA, Choi BY, Nam JH (2015). Association between living arrangements and depressive symptoms among older women and men in South Korea. Soc Psychiatry Psychiatr Epidemiol.

[35] Kim SH, Kim JS, Choi JY, Lee KY, Park TJ, Lee JH (2011). Factors Associated with Depressive Symptom in Chronic Obstructive Pulmonary Disease Based on the Third (2005) and Fourth (2008) Korea National Health and Nutritional Examination Survey (KNHANES III, IV). Korean J Family Med.

[36] China MoHotPsro (2011). The National Stardard of Basic Public Health Service (2011) (in Chinese). National Health and Family Planning Commission of the People's Republic of China. vol. 2014.

[37] Wee LE, Yong YZ, Chng MW, Chew SH, Cheng L, Chua QH (2014). Individual and area-level socioeconomic status and their association with depression amongst community-dwelling elderly in Singapore. Aging Mental Health.

[38] Lai DW (2009). Depressive symptoms of elderly Chinese in Guangzhou, Hong Kong, and Taipei. Aging Mental Health.

[39] Bekhet AK, Zauszniewski JA (2012). Mental health of elders in retirement communities: is loneliness a key factor?. Arch Psychiatr Nurs.

[40] Victor CR, Yang K (2012). The prevalence of loneliness among adults: a case study of the United Kingdom. J Psychol.

[41] Gerst-Emerson K, Shovali TE, Markides KS (2014). Loneliness among very old Mexican Americans: findings from the Hispanic Established Populations Epidemiologic Studies of the Elderly. Arch Gerontol Geriatr.

[42] Kanner AD, Coyne JC, Schaefer C, Lazarus RS (1981). Comparison of two modes of stress measurement: daily hassles and uplifts versus major life events. J Behav Med.

[43] Tiedt AD (2010). The gender gap in depressive symptoms among Japanese elders: evaluating social support and health as mediating factors. J Cross Cultural Gerontol.

[44] Christensen H, Jorm AF, Mackinnon AJ, Korten AE, Jacomb PA, Henderson AS (1999). Age differences in depression and anxiety symptoms: a structural equation modelling analysis of data from a general population sample. Psychol Med.

[45] Berry JM, Storandt M, Coyne A (1984). Age and sex differences in somatic complaints associated with depression. J Gerontol.

